# Identification of a Novel Variant in *MT-CO3* Causing MELAS

**DOI:** 10.3389/fgene.2021.638749

**Published:** 2021-05-12

**Authors:** Manting Xu, Robert Kopajtich, Matthias Elstner, Zhaoxia Wang, Zhimei Liu, Junling Wang, Holger Prokisch, Fang Fang

**Affiliations:** ^1^Department of Neurology, Beijing Children’s Hospital, Capital Medical University, National Center for Children’s Health, Beijing, China; ^2^Institute of Human Genetics, School of Medicine, Technical University of Munich, Munich, Germany; ^3^Institute of Neurogenomics, Helmholtz Zentrum München, Munich, Germany; ^4^Department of Neurology, School of Medicine, Technical University of Munich, Munich, Germany; ^5^Department of Neurology, Peking University First Hospital, Beijing, China

**Keywords:** MELAS, novel mitochondrial DNA variant, *MT-CO3* gene, complex IV of respiratory chain, mitochondrial diseases

## Abstract

Mitochondrial encephalomyopathy, lactic acidosis, and stroke-like episodes (MELAS) is a maternally inherited mitochondrial disease. Most cases of MELAS are caused by the m.3243A > G variant in the *MT-TL1* gene encoding tRNALeu^(UUR)^. However, the genetic cause in 10% of patients with MELAS is unknown. We investigated the pathogenicity of the novel mtDNA variant m.9396G > A/*MT-CO3* (p.E64K), which affects an extremely conserved amino acid in the CO3 subunit of mitochondrial respiratory chain (MRC) complex IV (CIV) in a patient with MELAS. Biochemical assays of a muscle biopsy confirmed remarkable CIV deficiency, and pathological examination showed ragged red fibers and generalized COX non-reactive muscle fibers. Transfer of the mutant mtDNA into cybrids impaired CIV assembly, followed by remarkable mitochondrial dysfunction and ROS production. Our findings highlight the pathogenicity of a novel m.9396G > A variant and extend the spectrum of pathogenic mtDNA variants.

## Introduction

Mitochondrial encephalomyopathy, lactic acidosis, and stroke-like episodes (MELAS) (OMIM 540000) is one of the most common maternally inherited mitochondrial disorders. Its clinical manifestations include stroke-like episodes, dementia, epilepsy, myopathy, recurrent headaches, hearing impairment, diabetes, and short stature (El-Hattab et al., 2015). The diagnostic criteria for MELAS include the presence of these typical manifestations and two of the following three indications of mitochondrial dysfunction: (i) a biochemical defect in mitochondrial energy metabolism, (ii) mitochondrial abnormalities determined by muscle biopsy, and (iii) a pathogenic variant associated with MELAS (Hirano et al., 1992; Yatsuga et al., 2012; Lorenzoni et al., 2015).

Mitochondrial encephalomyopathy, lactic acidosis, and stroke-like episodes is a genetically heterogeneous mitochondrial disorder (El-Hattab et al., 2015). The m.3243A > G variant in the *MT-TL1* gene encoding tRNALeu^(UUR)^ is found in approximately 80% of patients with MELAS. Other variants, such as m.3271T > C and m.3252A > G in *MT-TL1*, are also associated with MELAS. Moreover, numerous mitochondrial DNA (mtDNA) encoded genes have other rare variants that can cause MELAS; these include mtDNA encoded tRNAs (*MT-TS1, MT-TS2, MT-TW, MT-TC, MT-TL2, MT-TK, MT-TH, MT-TQ, MT-TF*, and *MT-TV*) and mitochondrial respiratory chain (MRC) complex I (*MT-ND1, MT-ND5*, and *MT-ND6*), complex III (*MT-CYB*), and complex IV (CIV) subunits (*MT-CO2* and *MT-CO3*). In addition, variants in nuclear genes, such as *POLG* and *FASTKD2*, have been associated with a MELAS-like phenotype (Cheldi et al., 2013). However, the molecular pathomechanisms of these variants are highly complex and far from well-documented, and in 10% of patients with MELAS the genetic cause is unknown (Cheldi et al., 2013; El-Hattab et al., 2015). Therefore, it is important to identify novel pathogenic variants and elucidate how they directly affect the function of mitochondria.

Here, we report a patient with MELAS carrying a novel m.9396G > A variant in *MT-CO3*. This is the fourth reported MELAS case with a variant of the genes encoding the CIV subunit. We confirmed the pathogenicity of this variant based on clinical characteristics, muscle pathology results, biochemical analyses, and transmitochondrial cytoplasmic hybrid (cybrid) cell studies.

## Subjects and Methods

### Clinical Description

The young female patient was the second child of Chinese non-consanguineous parents, born at full-term after a normal pregnancy (birth weight 3.5 kg, height 50 cm). Her early neurodevelopment was entirely normal. However, she often complained of fatigue and had short stature from about 3 years of age.

**FIGURE 1:**
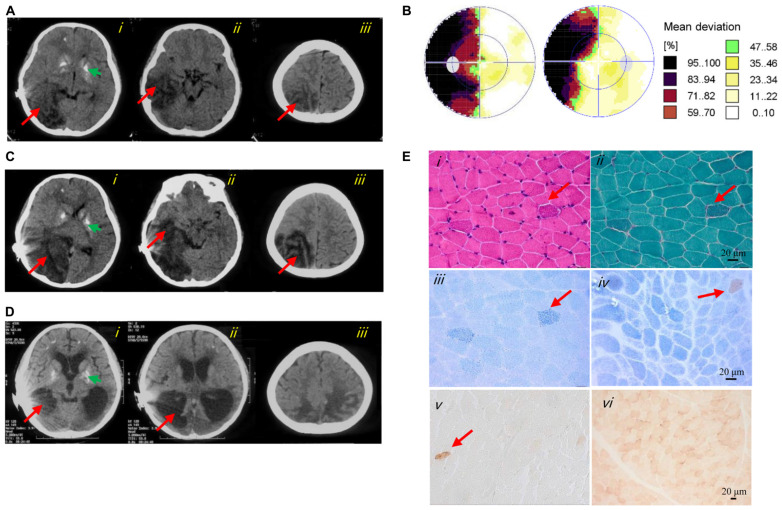
Clinical images of the patient. **(A)** Axial CT images of the first stroke-like episode revealed cerebral edema and brain herniation in the right occipital (*i*), temporal (*ii*), and parietal (*iii*) lobes (red arrows). The green arrow indicated basal ganglia calcification (*i*). **(B)** Perimetry showed left-side hemianopsia. **(C)** Axial CT images of the second stroke-like episode revealed more severe cerebral infarction and cerebral edema in the right occipital (*i*), temporal (*ii*), and parietal (*iii*) lobes, and in the right cerebellar hemisphere (*i*) (red arrows). The green arrow indicated basal ganglia calcification (*i*). **(D)** Axial CT images at 12 years of age showed cerebral atrophy (*i–iii*), an enlarged lateral cerebroventricle (*i–ii*) and multiple calcifications (*i*). **(E)** Image *i* showed several subsarcolemmal basophilic substance (red arrow; HE staining). Image *ii* showed several RRFs (red arrow; MGT staining). Image *iii* showed ragged blue fibers (red arrow; SDH reaction). Images *iv* and *v* showed general COX-deficient fibers (COX-SDH reaction and COX reaction, respectively). The red arrow in image *iv* and *v* indicated a COX-positive fiber. Image *vi* showed a normal muscle sample from an age matched control (COX reaction).

At 8 years of age, she developed a hearing impairment after a respiratory infection (never use aminoglycoside antibiotics) and was given a cochlear implant. After another infection at 9 years of age, she experienced a stroke-like episode that initially presented with frequent convulsions and headache, followed by visual impairment, vomiting, and gradual loss of consciousness. Cerebrospinal fluid lactate (8 mmol/L, normal range < 2.8 mmol/L) and serum lactate (10.7 mmol/L, normal range < 2.8 mmol/L) were elevated. All other laboratory results were normal, including blood cell counts, creatine kinase, serum glucose, ammonia, liver and kidney function tests, electrocardiogram, and echocardiography. Computed tomography (CT) showed large hypodense lesions in the right occipital, parietal, and temporal lobes, suggestive of cerebral edema and infarction. The midline of the occipital lobe was shifted slightly to the left, indicating brain herniation. Multiple calcifications in the bilateral basal ganglia were also evident on CT (Figure 1A). An electroencephalogram showed abnormally slow background activity in the occipital region and epileptiform discharges in both frontal and temporal regions. Perimetry presented left side hemianopsia (Figure 1B). Based on the clinical course, MELAS was suspected, and L-arginine, levetiracetam, and antioxidants were prescribed. After 23 days, she regained consciousness with no headache, convulsions, or visual impairment and was discharged from hospital.

One and a half months later, she experienced a similar stroke-like episode after fatigue, initially with a headache, vomiting, and left side hemianopsia, which progressed to unconsciousness. CT showed enlarged cerebral infarction lesions in the right occipital, parietal and temporal lobes, and right cerebellar hemisphere. More severe cerebral edema and cerebral herniation were also seen (Figure 1C). After treatment for several days, she improved, but the hemianopsia and hemiparesis remained.

The patient is now 12 years of age and undergoes continued treatment with levetiracetam and antioxidants. She still complains of intermittent headache and suffers from hemiparesis, dementia, poor emotional control, deafness, and hemianopsia. Repeat CT shows cerebral atrophy and multiple calcifications (Figure 1D). Her parents (both over 50 years of age) and 18-year-old brother are asymptomatic.

### Histopathological, Histochemical, and Biochemical Analyses of Muscle Biopsy

The right bicep of the 11-year-old patient was biopsied, and fresh specimens were prepared for standard histopathology staining with hematoxylin and eosin (HE), modified Gomori trichrome (MGT), and Oil Red O, as well as histochemical staining with succinate dehydrogenase (SDH), and COX-SDH reactions analyses (Parikh et al., 2015). The activities of the MRC complexes and the mitochondrial marker enzyme citrate synthase (CS) were evaluated in muscle homogenates as described previously (Ogawa et al., 2017).

### Molecular Genetic Analyses

Total DNA was extracted from peripheral blood, and mtDNA from skeletal muscle, skin fibroblasts, blood, urine, saliva, and fingernail samples, as described previously (Ji et al., 2014). Mitochondrial genome next-generation sequencing and whole-exome sequencing (WES) were performed to detect causative genes using an Illumina HiSeq X Ten sequencer (Illumina, San Diego, CA, United States). Sanger sequencing was conducted using 2 × Taq Master Mix (Thermo Fisher Scientific, Waltham, MA, United States) and Applied Biosystems thermal cyclers (Applied Biosystems, Foster City, CA, United States). Mutation loads were determined by quantitative pyrosequencing; quantification of the heteroplasmy level of each variant was achieved using PyroMark Q24 software (Grady et al., 2018).

The PolyPhen2 and PROVEAN were used to evaluate the pathogenicity of the mtDNA variants (Adzhubei et al., 2013; Choi and Chan, 2015). PyMOL software was used to visualize the protein structure and model amino acid changes in the three-dimensional structure.

### Generation of Transmitochondrial Cybrids

To generate transmitochondrial cybrids carrying the m.9396G > A variant, platelets were isolated from blood samples and fused with mtDNA-less human osteosarcoma 143B cells as described previously (Chomyn, 1996). The cybrid clones were cultured in Dulbecco’s modified Eagle’s medium (Thermo Fisher Scientific) supplemented with 1% penicillin and 10% fetal bovine serum (both Thermo Fisher Scientific) at 37°C in a 5% CO_2_ atmosphere. After hybridization, we selected cybrid clones with more than 95% mutant mtDNA, 78.6% mutant mtDNA, and those lacking this variant [wild-type (WT)] for further experiments.

### RNA Isolation and Quantitative Reverse-Transcription (qRT)-PCR

Total RNA was extracted using the TRIzol reagent (Invitrogen, Carlsbad, CA, United States) according to the manufacturer’s procedure, and reverse-transcribed into complementary DNA (cDNA) using a cDNA Reverse Transcription Kit (Applied Biosystems). Quantitative PCR was conducted with SYBR Green Master Mix (Applied Biosystems) on the 7500 Fast Real-Time PCR System (Applied Biosystems). The primer sequences for *MT-CO3* were as follows: sense, 5′-CAC ATC CGT ATT ACT CGC ATC-3′ and antisense, 5′-GAA GTA CTC TGA GGC TTG TAG-3′; the primers for *GAPDH* (internal control) were sense, 5′-CGC TGA GTA CGT CGT GGA GTC-3′ and antisense, 5′-GCT GAT GAT CTT GAG GCT GTT GTC-3′. The data were analyzed by the 2^–ΔΔ*Ct*^ method.

### Western Blots and Blue Native Polyacrylamide Gel Electrophoresis

Protein extraction from cybrids for sodium dodecyl sulfate (SDS)-polyacrylamide gel electrophoresis (PAGE) and Western blotting was carried out as described previously (Ji et al., 2020). Briefly, cells were lysed in radioimmunoprecipitation assay (RIPA) buffer (Sigma-Aldrich, St. Louis, MO, United States). The protein extracts were resolved in a 10% SDS polyacrylamide gel and transferred to a polyvinylidene difluoride membrane. Membranes were blocked with 5% milk for 1 h and incubated with the following primary antibodies at 4°C overnight: anti-MT-CO3 (cat #ab110259; Abcam, Cambridge, United Kingdom) and VDAC (cat #4661; Cell Signaling Technology, Danvers, MA, United States); the latter served as a loading control. Blue native (BN)-PAGE was performed with mitochondrial proteins isolated from cybrids as described previously (Delmiro et al., 2013), using antibodies against MT-COXIV (ab202554; Abcam), SDHA (ab14715; Abcam), VDAC (cat#4661; Cell Signaling Technology), UQCRC2 (ab14745; Abcam), NDUFS2 (ab110249; Abcam), and ATPB (ab14730; Abcam). The in-gel activity (IGA) of CIV was performed as described previously (Jha et al., 2016). Briefly, after running the native PAGE, incubate the gel in 20 ml of complex IV substrate. Appearance of brown bands is indicative of CIV activity.

### Measurement of Oxygen Consumption Rate and MRC Complex Activities of the Cybrids

Cybrid cells were seeded at a density of 4 × 10^4^ cells per well on Seahorse XFe24 polystyrene cell culture plates. The oxygen consumption rate (OCR) of the cybrids was assayed using the XF96 Extracellular Flux Analyzer (Agilent Technologies, Santa Clara, CA, United States), as described previously (Kremer and Prokisch, 2017; Ogawa et al., 2017). The activities of MRC complexes I, II, II + III, and IV and the mitochondrial marker enzyme CS were evaluated in cybrids as described previously (Ogawa et al., 2017).

### Assays of ATP and Reactive Oxygen Species

The ATP levels of cybrids were measured using the ATP Bioluminescence Assay Kit HS II (Roche, Indianapolis, IN, United States). Briefly, cybrids were incubated in a solution [156 mM NaCl, 3 mM KCl, 2 mM MgSO_4_, 1.25 mM KH_2_PO_4_, 2 mM CaCl_2_, 20 mM 4-(2-hydroxyethyl)-1-piperazineethanesulfonic acid (HEPES); pH 7.35] with glucose (total ATP production) or 2-deoxy-D-glucose (2-DG) plus pyruvate (oxidative ATP production). Relative mitochondrial ATP synthesis was defined as the ratio relative to total ATP production of cybrids WT.

Reactive oxygen species (ROS) levels were measured as described elsewhere (Wang et al., 2011). Briefly, approximately 1 × 10^6^ cybrids were harvested and suspended in phosphate-buffered saline (PBS) with 10 μM 2’,7’-dichlorodihydrofluorescein diacetate. After incubation (37°C, 5% CO_2_) for 20 min, the cybrids were washed and resuspended in PBS. Finally, the fluorescence signals of cybrids were determined using excitation and emission wavelengths of 488 and 529 nm, respectively.

### Statistical Analysis

Statistical parameters are reported in the figure legends. *In vitro* experiments were repeated at least twice. Statistical analysis was performed using Prism 8.0 for Windows (GraphPad software, Inc., San Diego, CA, United States). Differences between mutant and WT cybrid cells were evaluated by one-way analysis of variance (ANOVA) followed by the Tukey’s honestly significant difference *post hoc* test.

## Results

### Muscle Biopsy Analyses Revealed Mitochondrial Dysfunction and a CIV Defect

Muscle histopathology showed slightly increased variation in fiber size and subsarcolemmal basophilic substance on HE staining. Moderately increased fat deposition was noted in some fibers by Oil Red O staining. Ragged red fibers (RRFs) and ragged blue fibers were detected in 2–5% of the total biopsy on MGT staining and SDH reaction, suggestive of abnormal mitochondrial accumulation. Remarkable mitochondrial abnormalities were detected, characterized by COX deficiency affecting >90% of all fibers with the COX-SDH and COX reaction, respectively. In addition, a few arterioles were identified in the COX-deficient muscle section (Figure 1E). The activities of CIV in the patient’s muscles were reduced to 16.5 ± 6.5% and 20.3 ± 8.1% compared with those of CS and complex II, respectively (Table 1).

**TABLE 1:** MRC enzyme assays of the muscle biopsy.

	**CI**	**CII**	**CII + III**	**CIII**	**CIV**
% of CS	78.2 ± 2.9	77.1 ± 3.6	80.1 ± 8.1	64.9 ± 7.1	16.5 ± 6.5
% of CII	98.6 ± 6.1	−	100.8 ± 9.4	82.4 ± 6.0	20.3 ± 8.1

*Enzyme activities are expressed as percentages of normalized mean values relative to CS and CII. CI, complex I; CII, complex II; CIII, complex III; CIV, complex IV; CS, citrate synthase.*

### Identification of a Probably Pathological Variant in *MT-CO3*

Based on pathological and biochemical analyses, the patient was confirmed to have MELAS with CIV deficiency. Although no known pathogenic mtDNA variant was identified by sequencing mitochondrial genes, and there was no potential biallelic rare variant of any gene associated with mitochondrial disease, CIV subunits or assembly factors were detected by WES.

**FIGURE 2:**
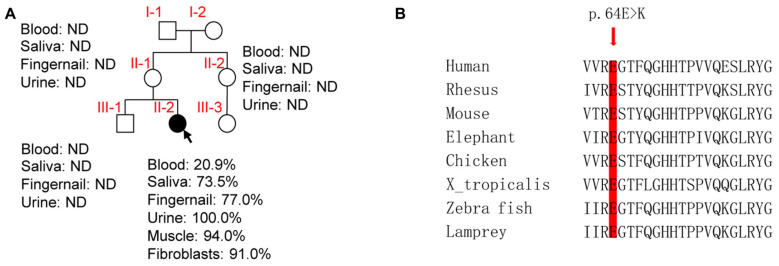
Molecular genetic assays. **(A)** Family pedigree of the patient (arrow indicates the proband). ND, not detected. **(B)** Phylogenetic conservation of the human MT-CO3. Arrow, p.64E > K.

The homoplasmic m.9396G > A (p.E64K) variant of *MT-CO3* was detected in the patient’s urinary sediment. Skeletal muscle, skin fibroblasts, saliva, fingernail and blood samples were screened for the presence of the m.9396G > A variant and exhibited heteroplasmy at this position of 94.0, 91.0, 73.5, 77.0, and 38.9%, respectively. The novel m.9396G > A variant was not detected in saliva, urinary sediment, fingernail or blood DNA samples from the patient’s mother, aunt, or older brother, implying that the variant had arisen *de novo* during embryogenesis and was not maternally-inherited (Figure 2A). No other suspected pathogenic variant was identified in the mtDNA (Supplementary Table S1), as all other base substitutions represented mtDNA polymorphisms in the MITOMAP database^1^.

**FIGURE 3:**
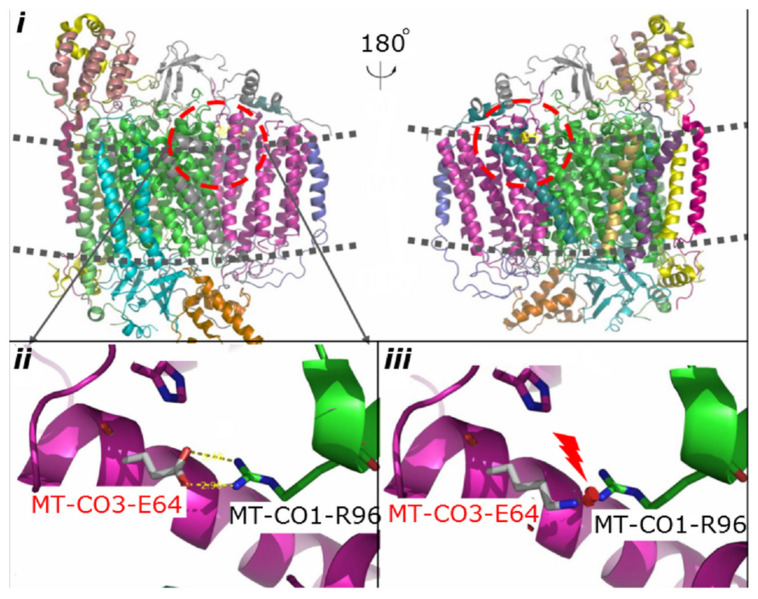
Predicted structural model of MT-CO3. Image *i* is an overview of complex IV. Image *ii* shows the hydrogen bond between the WT glutamate at position 64 of MT-CO3 and an arginine in MT-CO1. Image *iii* shows the broken hydrogen bond caused by the change from glutamate to lysine at position 64 of MT-CO3.

Phylogenetic conservation analysis of the p.64 aminoacidic residue in eight species (human, rhesus, mouse, elephant, chicken, X. tropicalis, zebrafish, and lamprey) revealed that this position is highly conserved (Figure 2B). The PolyPhen2 score was 1.000, predicted to be “damaging.” The PROVEAN score was -3.72, predicted to have a “deleterious” effect on the protein’s function. The predicted structure revealed that the MT-CO3 protein was not truncated, but the stable combination of MT-CO3 with MT-CO1 was probably altered due to destruction of the hydrogen bond between the evolutionary conserved glutamate of MT-CO3 at position 64 and an arginine of MT-CO1 at position 96, resulting in incomplete assembly and dysfunction of CIV (Figure 3).

### Effect of the m.9396G > A Variant on Transcript and Protein Levels

**FIGURE 4:**
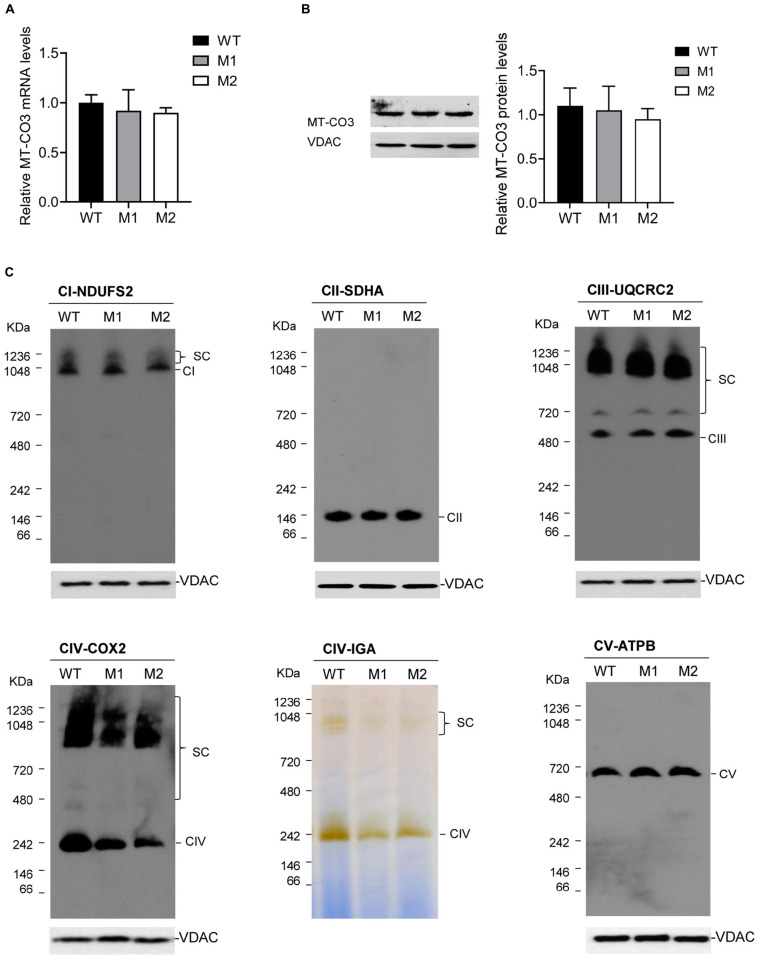
Effect of the m.9396G > A variant on transcript and protein levels. **(A)** Relative *MT-CO3* mRNAs levels in cybrids M1 and M2 relative to that in WT cybrids. **(B)** Relative MT-CO3 protein levels in cybrids WT, M1, and M2 determined by Western blotting. VDAC was used as the internal control. **(C)** For cybrids WT, M1, and M2, IGA analysis of CIV and BN-PAGE/immunoblot analysis of MRC complexes using antibodies against subunits of complexes I (NDUFS2), II (SDHB), III (UQCRC2), IV (COXIV), and V (ATPB). VDAC was used as the internal control. CI, complex I; CII, complex II; CIII, complex III; CV, complex V; SC, supercomplex.

qRT-PCR analysis showed almost equal *MT-CO3* mRNA levels between two cybrid clones with more than 95% mutant mtDNA (M1 and M2) and one without this variant (WT) (Figure 4A). Consistently, Western blotting showed almost equal MT-CO3 protein levels in cybrids M1 and M2 compared with WT cybrids (Figure 4B).

The steady-state levels of MRC complexes were determined by BN-PAGE. The amount of mature CIV and the IGA of CIV in M1 and M2 clones were significantly reduced compared with the WT cybrids (Figure 4C), consistent with the CIV enzymatic defect detected in the patient. However, all other complexes exhibited similar expression levels in M1 and M2 clones compared with the WT cybrids.

### The m.9396G > A Variant Results in Mitochondrial Dysfunction

**FIGURE 5:**
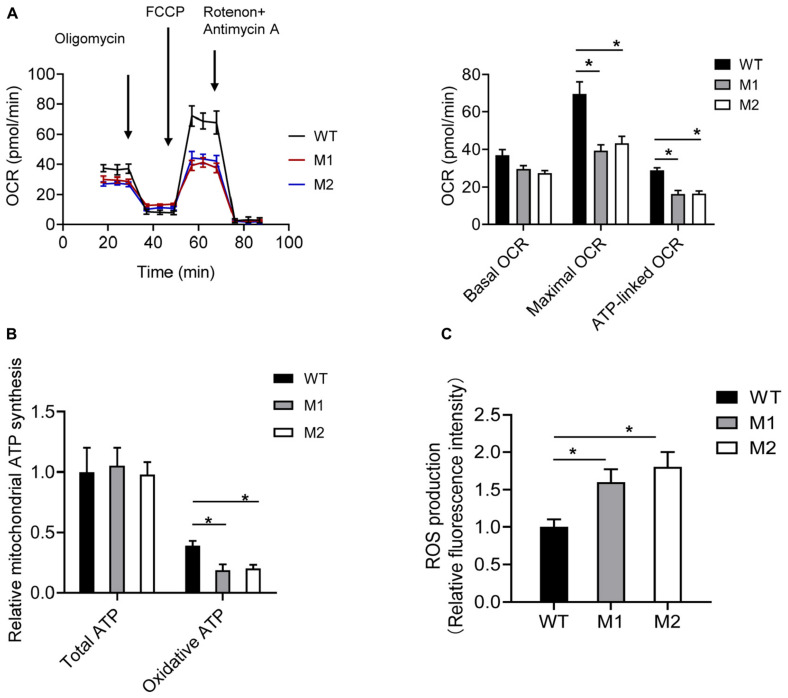
The m.9396G > A variant results in mitochondrial dysfunction. **(A)** Analysis of OCR in WT (black), M1 (blue), and M2 (red) cybrids using the inhibitors oligomycin, FCCP, antimycin A, and rotenone. The basal OCR, maximal OCR, and ATP-linked OCR in cybrids WT, M1, and M2 are shown. Values are means ± SD. *p < 0.05 by one-way ANOVA. **(B)** Total and oxidative ATP levels in cybrids WT, M1, and M2. Values are means ± SD. *p < 0.05 by one-way ANOVA. **(C)** ROS production in cybrids WT, M1, and M2. Values are means ± SD. *p < 0.05 by one-way ANOVA.

The basal OCR of cybrids M1 and M2 was 79.9 and 73.7% relative to the WT cybrids, respectively. The ATP-linked OCR in cybrids M1 and M2 was significantly reduced, to 56.8 and 57.2%, respectively, of that in WT cybrid cells. The maximal OCR in the two mutant cybrid clones was significantly reduced to 56.5 and 62.2%, respectively, compared with the value in WT cybrids (Figure 5A). Biochemical assays were performed to determine the activities of MRC complexes. Cybrids M1 and M2 exhibited a significant reduction in CIV activity compared with the WT cybrids. However, no significant difference was observed in complex I, II, II + III, or III activities between the mutant and WT cybrids (Table 2).

**TABLE 2:** MRC enzyme assays in WT and mutant cybrids.

	**WT cybrids**	**Mutant cybrids**	
		**M1**	**M2**
CI/CS	83.3 ± 8.1	76.1 ± 7.4	80.5 ± 10.9
CII/CS	70.9 ± 1.6	65.3 ± 15.2	57.1 ± 12.5
CII + III/CS	83.1 ± 10.7	91.9 ± 8.5	84.3 ± 10.2
CIII/CS	107.2 ± 16.1	114.2 ± 13.6	119.6 ± 10.2
CIV/CS	86.4 ± 14.0	36.3 ± 10.5	41.5 ± 7.3

*Enzyme activities are expressed as percentages of normalized mean values relative to CS. CI, complex I; CII, complex II; CIII, complex III; CIV, complex IV; CS, citrate synthase.*

Cybrids were cultured in glucose or 2-DG and pyruvate. The ATP levels of cybrids M1 and M2 were similar to those of the WT cybrids in glucose medium. However, in 2-DG and pyruvate medium, the ATP levels were significantly reduced in cybrids M1 and M2. Relative mitochondrial ATP production was 39.1% in the WT cybrids compared to 18.5 and 20.1% in the mutant cybrids (Figure 5B). Moreover, the levels of mitochondrial ROS production in cybrids M1 and M2 were significantly increased, by 1.61- and 1.84-fold, relative to the WT cybrids (Figure 5C).

A cybrid clone with the mutation load of 78.6% for m.9396G > A variant (M3) was also selected for ATP and ROS production experiments. The ATP levels of cybrids M3 were similar to those of WT cybrids in glucose medium, while significantly reduced in 2-DG and pyruvate medium. Relative mitochondrial ATP production was 48.1% in the WT cybrids compared to 31.4% in cybrid M3 (Supplementary Figure S1A). Moreover, the levels of mitochondrial ROS production in cybrids M3 was significantly increased, by 1.45-fold, relative to the WT cybrids (Supplementary Figure S1B).

## Discussion

Here, we describe a young female patient who met the diagnostic criteria of MELAS (Hirano et al., 1992; Yatsuga et al., 2012). The patient presented with typical manifestations of MELAS, including recurrent stroke-like episodes, seizures, visual impairment, hemiparesis, exercise intolerance, short stature, deafness and intermittent headache. She also had a variety of biochemical defects in mitochondrial energy metabolism, including an increased lactate level in cerebrospinal fluid and blood, and CIV activity deficiency in muscle. Finally, she exhibited mitochondrial pathological abnormalities including RRFs and COX-deficient fibers on muscle biopsy.

Whole-exome sequencing revealed no potential rare biallelic variant of genes for any known diseases, CIV subunits, or assembly factors. By mtDNA sequencing, only the novel m.9396G > A variant was detected. The evidence supporting the pathogenicity of this variant is as follows. This variant is not listed as a single-nucleotide polymorphism in the MITOMAP database^2^, and was not detected in our Chinese (>900 mtDNA sequences) and European (>20,507 mtDNA sequences) in-house database. This variant was nearly homoplasmic in clinically affected skeletal muscle, but only presented 38.9% in blood. The variant arose *de novo* and was not detected in the patient’s clinically unaffected mother or maternal relatives. The variant resulted in substitution of a highly evolutionarily conserved glutamate at position 64 with a lysine, which probably affected the assembly of MT-CO3 with other CIV subunits and resulted in CIV dysfunction, as predicted *in silico*. Marked CIV deficiency was indicated by the results of biochemical assays, and generalized COX non-reactive muscle fibers were detected in histochemical assays, which were consistent with the location of the variant in a gene encoding one of the CIV subunits. Variants in *MT-CO3* are reportedly associated with MELAS (Rossmanith et al., 2008). The most important evidence for this is the marked reduction of mature CIV and mitochondrial dysfunction in the mutant cybrid cells, excluding nuclear variants and confirming the pathogenicity of the m.9396 G > A variant.

Although MELAS is a common mitochondrial disorder in which many pathogenic mtDNA variants have been reported, MELAS caused by variants in CIV encoding genes is rare (Manfredi et al., 1995; Choi et al., 2008; Rossmanith et al., 2008). Only three other cases have been reported previously, including one MELAS case with m.7630delT in *MT-CO2* and two with m.9957T > C in *MT-CO3* (Supplementary Table S2). The clinical and neuroradiological findings of these cases were characteristic. The pathological results of a muscle biopsy, however, were more distinctive than normal for MELAS, as first described by Rossmanith (Manfredi et al., 1995; Choi et al., 2008; Rossmanith et al., 2008). The muscle biopsy showed pronounced COX-negative fibers, which are less common in typical MELAS phenotypes (B. O. Manfredi et al., 1995; Choi et al., 2008; Rossmanith et al., 2008). Furthermore, only a few RRFs were seen, whereas the proportion of RRFs is usually high in 3243A > G MELAS (Manfredi et al., 1995; Choi et al., 2008; Rossmanith et al., 2008). In addition, intramuscular arteries, which are typically COX-positive in 3243A > G MELAS, are COX-negative in MELAS patients with a genetic CIV deficiency (Manfredi et al., 1995; Choi et al., 2008; Rossmanith et al., 2008). Our patient was clinically compatible with typical MELAS and pathologically consistent with the condition discussed by Rossmanith et al. (2008), supporting the pathological muscle peculiarities of rare MELAS caused by deleterious variants in the essential CIV subunit gene.

Complex IV in humans is composed of 14 subunits, including 3 core subunits (MT-CO1, MT-CO2, and MT-CO3) encoded by mtDNA, and 11 accessory subunits encoded by nuclear DNA. MT-CO3, with seven transmembrane helices, stabilizes the other two core subunits and acts as an initial proton acceptor in one of the transfer pathways. Compatibly, the m.9396G > A variant, located in one of the highly conserved transmembrane helices of MT-CO3, impairs the synthesis of CIV as studied in patient’s mutant cybrids, leading to mitochondrial dysfunction. The ATP-linked and maximal OCR were significantly reduced in the mutant cybrids compared with the WT cybrids. The maximal OCR may allow cells to protect mitochondria from oxidative insults. Impairment of OCR promotes electron leakage, elevating the oxidative stress level. Consistently, ROS production in cybrids carrying this variant was significantly increased compared with that in WT cybrids. Mutant cybrids were unable to proliferate normally on 2-DG medium, indicating a deleterious effect of the variant on viability when cells were forced to obtain ATP from the OXPHOS system. The mitochondrial dysfunction mentioned above may have contributed to the pathogenesis of MELAS in this patient.

## Conclusion

We report a patient with a rare form of MELAS associated with the novel m.9396G > A variant in *MT-CO3* and demonstrated the molecular pathomechanisms of this variant, extending the spectrum of pathogenic variants of this gene.

## Data Availability Statement

The datasets presented in this study can be found in online repositories. The names of the repository/repositories and accession number(s) can be found below: Genome Sequence Archive in National Genomics Data Center, accession no: GVM000119.

## Ethics Statement

The studies involving human participants were reviewed and approved by the Beijing Children’s Hospital. Written informed consent to participate in this study was provided by the participants’ legal guardian/next of kin.

## Author Contributions

MX contributed to the drafting of the original manuscript. FF contributed to the study concept and design, and critical revision of the manuscript for important intellectual content. All authors contributed to the acquisition and interpretation of data, and critical revision of the manuscript.

## Conflict of Interest

The authors declare that the research was conducted in the absence of any commercial or financial relationships that could be construed as a potential conflict of interest. The reviewer KM declared a past co-authorship with the authors HP and FF to the handling editor.

## Footnotes

1 http://www.mitomap.org/

2 http://www.mitomap.org/

## Supplementary Material

Supplementary Figure 1 The cybrid clone with a mutation load of 78.6% for m.9396G > A variant (M3) results in mitochondrial dysfunction. **(A)** Total and oxidative ATP levels in cybrids WT and M3. Values are means ± SD. ^∗^*p* < 0.05 by one-way ANOVA. **(B)** ROS production in cybrids WT and M3. Values are means ± SD. ^∗^*p* < 0.05 by one-way ANOVA.

Supplementary Table 1 List of all the variants in mtDNA sequencing for the patient.

Supplementary Table 2 A summary of reported patients with MELAS with pathogenic variants in the mitochondrial genes encoding CIV subunits.
