# Supplementary figures and images for: Identification of a Novel Variant in MT-CO3 Causing MELAS
Source: Front Genet. 2021 May 12;12:638749. doi: 10.3389/fgene.2021.638749 (PMC8153374; doi:10.3389/fgene.2021.638749)

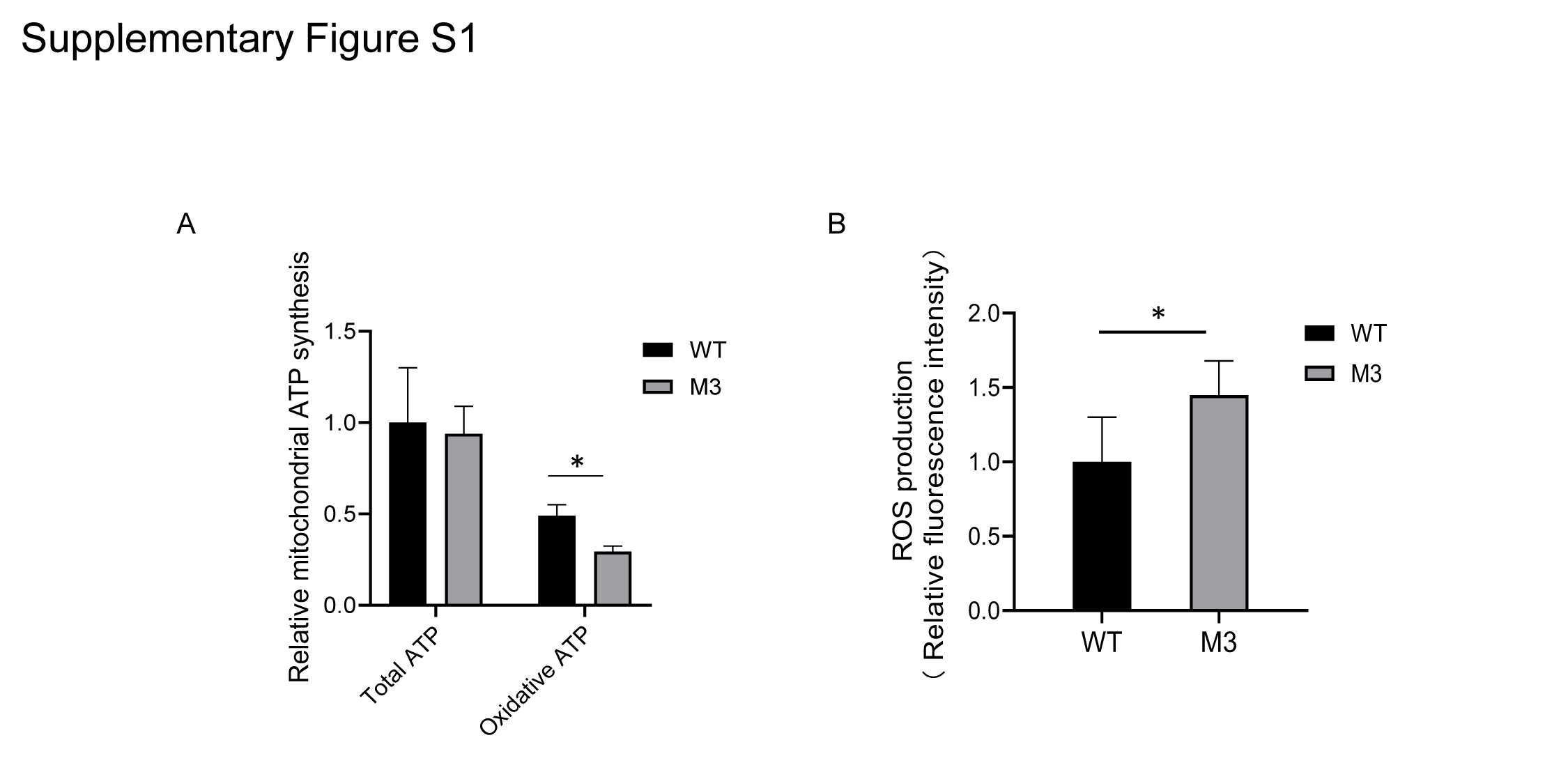

Supplement: Supplementary Figure 1 — The cybrid clone with a mutation load of 78.6% for m.9396G > A variant (M3) results in mitochondrial dysfunction. (A) Total and oxidative ATP levels in cybrids WT and M3. Values are means ± SD. ∗p < 0.05 by one-way ANOVA. (B) ROS production in cybrids WT and M3. Values are means ± SD. ∗p < 0.05 by one-way ANOVA. [file Image_1.TIF]
