# Supplementary material for: Identification of a Novel Variant in MT-CO3 Causing MELAS
Source: Front Genet. 2021 May 12;12:638749. doi: 10.3389/fgene.2021.638749 (PMC8153374; doi:10.3389/fgene.2021.638749)
Supplement: Supplementary Table 1 — List of all the variants in mtDNA sequencing for the patient. [file Table_1.DOCX]

**Table S1.** List of all the variants in mtDNA sequencing for the patient.

| No. | Variants | Genes | Variant types | Related disease | Mitomap Frequency | Sequencing depth | mutation loads (%) |
| --- | --- | --- | --- | --- | --- | --- | --- |
| 1 | m.9396G>A | MT-CO3 | missense |  |  | 102 | 21 |
| 2 | m.7250A>G | MT-CO1 | synonymous |  | 0.001 | 73 | 100 |
| 3 | m.9540T>C | MT-CO3 | synonymous |  | 0.309 | 78 | 100 |
| 4 | m.14659C>T | MT-ND6 | synonymous |  | 0.004 | 55 | 96 |
| 5 | m.4140C>T | MT-ND1 | synonymous |  | 0.001 | 67 | 99 |
| 6 | m.15040C>T | MT-CYB | synonymous |  | 0.002 | 128 | 100 |
| 7 | m.12549C>T | MT-ND5 | synonymous |  | 0.001 | 83 | 100 |
| 8 | m.13152A>G | MT-ND5 | synonymous |  | 0.001 | 110 | 100 |
| 9 | m.3167insC | MT-RNR2 | noncoding |  |  | 53 | 100 |
| 10 | m.12940G>A | MT-ND5 | missense |  | 0.007 | 123 | 99 |
| 11 | m.15071T>C | MT-CYB | missense |  | 0.002 | 122 | 100 |
| 12 | m.8793T>C | MT-ATP6 | synonymous |  | 0.004 | 67 | 100 |
| 13 | m.5483T>C | MT-ND2 | synonymous |  | 0.002 | 36 | 100 |
| 14 | m.14311T>C | MT-ND6 | synonymous |  | 0.001 | 83 | 100 |
| 15 | m.8856G>A | MT-ATP6 | synonymous |  | 0.004 | 62 | 100 |
| 16 | m.567insCCCCCC | MT-TF | intergenic |  | 0.001 | 8 | 100 |
| 17 | m.16271T>C | MT-TP | intergenic |  | 0.007 | 118 | 100 |
| 18 | m.10646G>A | MT-ND4L | synonymous |  | 0.003 | 85 | 100 |
| 19 | m.16086T>C | MT-TP | intergenic |  | 0.021 | 86 | 81 |
| 20 | m.10400C>T | MT-ND3 | synonymous |  | 0.206 | 44 | 100 |
| 21 | m.15218A>G | MT-CYB | missense |  | 0.021 | 109 | 100 |
| 22 | m.14783T>C | MT-CYB | synonymous |  | 0.205 | 63 | 100 |
| 23 | m.15301G>A | MT-CYB | synonymous |  | 0.263 | 83 | 100 |
| 24 | m.16298T>C | MT-TP | intergenic |  | 0.062 | 121 | 100 |
| 25 | m.10873T>C | MT-ND4 | synonymous |  | 0.309 | 49 | 100 |
| 26 | m.8701A>G | MT-ATP6 | missense |  | 0.309 | 80 | 100 |
| 27 | m.15043G>A | MT-CYB | synonymous |  | 0.228 | 131 | 100 |
| 28 | m.489T>C | MT-TF | intergenic |  | 0.253 | 37 | 100 |
| 29 | m.12705C>T | MT-ND5 | synonymous |  | 0.395 | 89 | 99 |
| 30 | m.16223C>T | MT-TP | intergenic |  | 0.365 | 109 | 100 |
| 31 | m.709G>A | MT-RNR1 | noncoding |  | 0.131 | 96 | 100 |
| 32 | m.16311T>C | MT-TP | intergenic |  | 0.194 | 125 | 99 |
| 33 | m.10398A>G | MT-ND3 | missense | longevity/ altered cell pH/ metabolic syndrome/ breast cancer risk | 0.421 | 43 | 100 |
