# Supplementary material for: Identification of a Novel Variant in MT-CO3 Causing MELAS
Source: Front Genet. 2021 May 12;12:638749. doi: 10.3389/fgene.2021.638749 (PMC8153374; doi:10.3389/fgene.2021.638749)
Supplement: Supplementary Table 2 — A summary of reported patients with MELAS with pathogenic variants in the mitochondrial genes encoding CIV subunits. [file Table_2.docx]

**Tabel S2. A summary of reported patients with MELAS with pathogenic variants in the mitochondrial genes encoding CIV subunits**.

| Gene | *MT-CO2* |  | *MT-CO3* | | |
| --- | --- | --- | --- | --- | --- |
| Variant | m. 77630delT |  | m. T9957C | m. T9957C | m. G9396A |
|  | - |  | p. F251L | p. F251L | p. E64K |
| Homo/heteroplasmy | heteroplasmy |  | heteroplasmy | homoplasmy | nearly homoplasmy |
| mutation loads in affected tissues | 93% in muscle |  | - | 100% in muscle | 94% in muscle |
| Mutation loads in blood | 28% |  | 60% | 100% | - |
| Literature citation | W Rossmanith(2008) |  | G Manfredi (1995) | Byung-Ok Choi (2008) | Current (2020) |
| Phenotype | MELAS |  | MELAS | MELAS | MELAS |
| **Clinical findings** |  |  |  |  |  |
| Age at onset | 13 years |  | 7 years | 25 years | 9 years |
| Sex | Male |  | Male | Male | Female |
| Stroke-like episode | + |  | + | + | + |
| Exercise intolerance | + |  | - | - | + |
| Progressive mental impairment | + |  | + | + | + |
| Short stature | + |  | - | + | + |
| Seizures | + |  | + | - | + |
| Cardiac abnormalities | left ventricular hypertrophy | | + | + | - |
| Ophthalmology | retinopathy |  | blindness,  mild ptosis | cataracts | - |
| Hearing loss | - |  | + | - | + |
| Endocrine abnormalities | - |  | - | diabetes, hypothyroidism | - |
| Nephropathy | + |  | - | - | - |
| Headaches and vomiting | + |  | - | - | + |
| Elevated lactic acid | + (serum) |  | + (serum and CSF) | + (serum) | + (serum and CSF) |
| Brain MRI | cerebral infarction |  | cerebral infarction | cerebral infarction | cerebral infarction |
| **Muscle biopsy** |  |  |  |  |  |
| RRF | 2% |  | a few | a few | 2-5% |
| COX-reaction | generalised COX negative |  | generalised COX positive | ND | generalised COX negative |
| Intramuscular arteries | COX-negative |  | ND | ND | COX-negative |
| Complex IV deficiency | + |  | - | ND | + |

ND: not determined
